# Towards a comprehensive school food environment audit tool in Canada: a systematic review of school food environment measurements and nutrition determinants

**DOI:** 10.1186/s12889-025-24937-w

**Published:** 2025-10-28

**Authors:** Hayun Jeong, Emma Wedekind, Dilothi Selvarajah, Mavra Ahmed, Chelsea McPherson, Daniel W. Sellen, Mary R. L’Abbe

**Affiliations:** 1 Department of Nutritional Sciences, Temerty Faculty of Medicine, University of Toronto, 1 Kings College Circle, Toronto, ON M5S 1A8 Canada; 2 Dalla Lana School of Public Health, University of Toronto, 155 College St, Toronto, ON M5T 3M6 Canada; 3 Faculty of Health Sciences, Ontario Tech University, 2000 Simcoe St N, Oshawa, ON L1G 0C5 Canada; 4 Joannah & Brian Lawson Centre for Child Nutrition, University of Toronto, 1 Kings College Circle, Toronto, ON M5S 1A8 Canada

**Keywords:** School food environment, Nutrition, Child health, Meal, Obesity, Measurement tools

## Abstract

**Background:**

School food programs (SFPs) support children’s health, learning, and well-being, yet Canada remains one of the few high-income countries without a nationally coordinated SFP. Instead, a patchwork of independent programs has created disparities in access, funding, and quality. In 2024, the federal government released Canada’s first National School Food Policy built on principles of accessibility, health promotion, inclusivity, flexibility, sustainability, and accountability, and committed $1 billion over five years. However, no clear frameworks exist for implementation or evaluation. This review examines existing measurement tools to identify captured dimensions of school food environments and student nutrition determinants, and assess alignment with Canada’s National School Food Policy to inform the development of a comprehensive monitoring and evaluation tool for Canada’s forthcoming national SFP.

**Methods:**

A systematic search of peer reviewed literature published prior to 2024 was conducted to identify measurement tools used to assess school food environments. Tools were categorized using three complementary frameworks: INFORMAS (food environment), the CDC School Nutrition Environment Framework (school policies/practices), and the Graziose Framework (student behaviors). A sub-analysis of Canadian tools assessed alignment with the National School Food Policy.

**Results:**

Of 695 articles screened, 101 met the inclusion criteria. Most tools used quantitative methods (61%), while others used qualitative (15%) or mixed (34%) methods. No single tool captured all relevant dimensions of school food environments or factors influencing students’ nutrition behaviors. The physical dimension was most commonly captured (92%) and the economic dimension the least (26%). School meals (75%) and Smart snacks (60%) were commonly measured, while staff role modelling was rarely included (10%). Most studies measured school policy (83%) and meal-specific factors (72%). Of the 7 Canadian tools, none captured all six principles of Canada’s National School Food Policy. Most tools were rated as ‘medium’ (48%) or ‘low’ quality (35%).

**Conclusions:**

Existing tools show methodological gaps and are limited coverage, highlighting the need for more comprehensive and high-quality audit tools. For Canada, such a tool must also capture all six principles of the National School Food Policy to support implementation, evaluation, and accountability of the forthcoming national SFP.

**Trial registration:**

CRD42023492602

**Supplementary Information:**

The online version contains supplementary material available at 10.1186/s12889-025-24937-w.

## Background

Renewed focus on national school food programs (SFPs) as a policy priority in recent years is based on evidence that schools serve as focal points for interventions that promote children’s health, academic success, and overall well-being [1]. Research has shown that well-implemented SFPs benefit children in multiple ways that include improved school attendance and academic performance [2–4], enhanced children’s access to and consumption of nutritious foods [4–6], and reduced risk of chronic diseases in adulthood [7, 8]. Beyond their direct positive impacts on students, successful SFPs contribute to broader societal gains, including reduced burden and cost of illness [9], improved health equity [10, 11], strengthened local food systems [9, 10], economic opportunities for farmers [9, 12], economic growth with a return of some nine dollars for every dollar invested [13], and progress toward the United Nations’ Sustainable Development Goals [10, 14]. However, for SFPs to fully realize these benefits, they must be designed to ensure broad accessibility [11], such as being universal or having minimal barriers to participation. Programs that are poorly funded, restrictive, or stigmatized may fail to reach the children who need them most [15], limiting their overall effectiveness.

Despite these benefits, Canada remained until 2024 among the few high-income countries without a nationally coordinated and funded SFP [16]. Currently, Canada continues to rely on a historically emergent patchwork of independently implemented programs across provinces, regions, and school boards [17, 18], leading to significant disparities in capacity, funding, and program quality. This fragmented approach creates inequities [17], leaving many children without consistent access to nutritious meals at school and missing out on the broader benefits of well-established SFPs. International examples, such as Finland and Brazil, demonstrate that structured, universally accessible SFPs not only improve children’s nutrition, academic performance, and food-related habits but also support local economies and job creation [19–22]. To align with global best practices, there is an unmet need across Canada for a commonly adopted set of indicators that can benchmark a comprehensive, equitable, and sustainable national SFP that ensures healthy meals and food literacy education for all children.

In response to the pressing interest and call for a national SFP, the federal government announced a commitment of $1 billion over five years in the 2024 budget to develop and implement Canada’s national SFP, with the goal of reaching up to 400,000 additional children annually [23]. In June 2024, the National School Food Policy was released, outlining the federal government’s long-term vision that all children and youth in Canada have access to nutritious, stigma-free meals at school while fostering healthy eating habits and strengthening local food systems [10]. This vision is guided by six principles: *accessibility*, *health promotion*, *inclusivity*, *flexibility*, *sustainability*, and *accountability* [10]. Beyond these principles, there are also clear objectives, such as progressively working toward universal access, prioritizing underserved and food-insecure communities, reducing barriers to participation, and supporting sustainable operations through infrastructure and staffing investments [10]. The policy also emphasizes fostering food literacy, promoting culturally appropriate programming, and creating opportunities to connect with local food systems. Together, these principles and objectives provide a roadmap for action but lack concrete steps for implementation, monitoring, or evaluation, raising concerns that this vision may remain aspirational rather than actionable.

A significant gap in the development of Canada’s national SFP is the absence of a robust monitoring and evaluation (M&E) framework. M&E must be integrated at every stage—from development to implementation and impact assessment—to ensure continuous improvement and accountability [24]. Without a standardized M&E framework and tool, assessing the success of SFPs and making evidence-based improvements remain, therefore, challenging. Various measurement tools, such as questionnaires and menu audits, exist to evaluate school food environments, yet each prioritizes specific outcomes, limiting their ability to provide a comprehensive assessment [25]. While previous reviews have explored and categorized these tools [25], none have systematically examined them in the context of informing the development of an M&E framework and tool that can guide the design and implementation of Canada’s national SFP.

To address this gap, we systematically reviewed the literature on school food environment measurement tools to: (1) identify the dimensions of school food environments and determinants of student nutrition behaviors captured by existing tools; (2) assess how well current tools align with the key principles outlined in Canada’s National School Food Policy; and (3) evaluate the quality of existing tools to inform the development of a M&E framework and subsequently a comprehensive audit tool for Canada’s future national SFP.

## Materials and methods

This systematic review followed the Preferred Reporting Items for Systematic Reviews and Meta-Analysis (PRISMA) guidelines [26] and was registered with the International Prospective Register of Systematic Reviews (CRD42023492602).

### Search strategy

This review builds upon search methods outlined in previous studies from O’Halloran et al. [25], and Lytle & Sokol [27]. An electronic search of peer-reviewed literature was first conducted in June and July of 2021 as part of an internal review that was not published. With the release of Canada’s National School Food Policy, an updated search was conducted in August 2024 to ensure the most recent literature was captured. The databases used for this systematic review were MEDLINE, Embase, the Cochrane Library, Scopus, and Web of Science.

Search terms were established to reflect three research concepts: (1) food environments; (2) schools; and (3) measurement tools. Initial search terms returned broad results and were thus refined to focus the scope of the results to relevant studies. Final search terms and search strategy is presented in Supplementary Material 1.

### Inclusion and exclusion criteria

Inclusion and exclusion criteria used in a previous review [25] served as the basis for our eligibility criteria. Studies included in this review were limited to those that were: (1) published in English; (2) human studies; (3) studies that specified the methods or tools used to assess the school food environments; (4) studies of elementary/primary schools, middle schools, and/or secondary/high schools. Since this review focused on school food environments, studies conducted in settings other than schools, such as day care centres, were excluded.

### Study selection

**Fig. 1:**
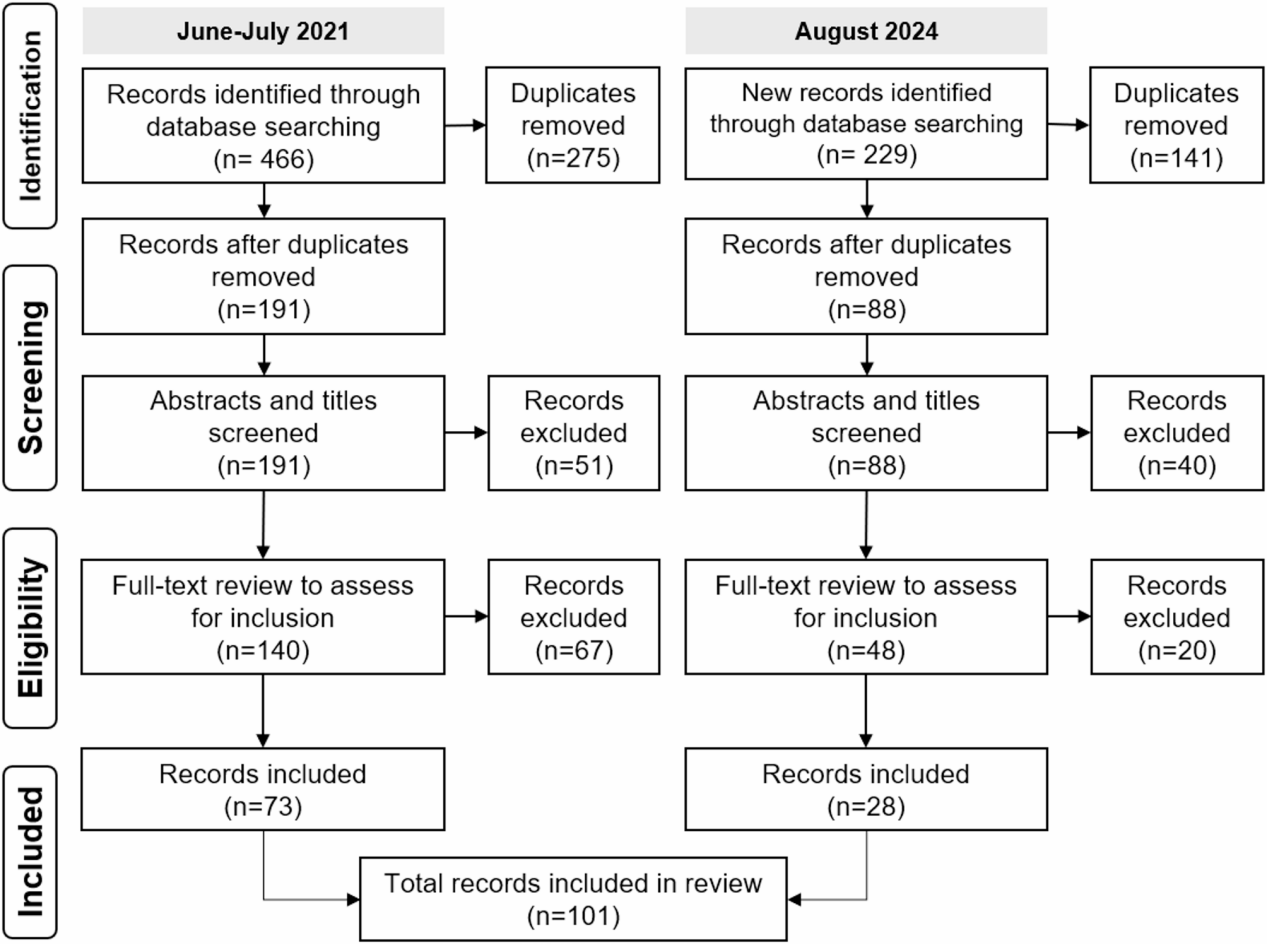
Preferred Reporting Items for Systematic Reviews and Meta-Analyses (PRISMA) flowchart summarizing the literature search process

The study selection process was performed manually using Microsoft Excel (Version 2507, Microsoft 365 MSO) by H.J., E.W., D.S., and M.A. The search results from all databases produced 466 records in 2021 and 229 records in 2024, for a total of 695 records. All records were reviewed and after 416 duplicates were removed, 279 abstracts and titles were screened by H.J. and E.W. to determine inclusion or exclusion of studies based on the specified search criteria. A total of 188 records that met the criteria from the first screening were then reviewed by M.A. and D.S., before undergoing a full-text review by H.J., E.W., D.S., and M.A. for a final selection based on their eligibility. In total, 101 articles met inclusion criteria and were selected for this review (Fig. 1).

### Frameworks for evaluating identified measurement tools

To comprehensively evaluate the school food environment, we applied three complementary frameworks that together provide a robust approach to assessing both the structural and behavioral influences on student nutrition (Table 1). The primary framework, Swinburn and colleagues’ INFORMAS (International Network for Food and Obesity/non-communicable Diseases Research, Monitoring and Action Support) food environment framework was chosen for its well-established methodology in evaluating food environments’ obesogenicity [28]. However, since the INFORMAS framework is not specific to school settings, we incorporated two additional frameworks (Centers for Disease Control and Prevention (CDC) Framework for Addressing School Nutrition Environment [29] and the Socioecological Framework of Environmental Influences [30] to provide a more granular and school-specific lens. While other frameworks (e.g., WHO Health Promoting Schools Framework) were considered, their broader whole-school health focus and limited detail on food environment and nutrition-specific dimensions made them less suitable for the current study objectives. Adaptations were made to these frameworks a priori to align them with the Canadian school context. Each tool identified from the included studies was coded against the three frameworks using a binary system (captured/not captured). Overall, the integration of these three frameworks enabled a multidimensional evaluation of school food environments in a way that is both evidence-based and aligned with Canada’s policy context, while also capturing the behavioral and socio-cultural influences that shape student nutrition outcomes. Importantly, the frameworks were mapped to the principles and objectives of Canada’s National School Food Policy [10] to examine how well existing tools capture priority areas for Canada’s forthcoming national SFP (Table 1).

**Table 1:** Alignment of the INFORMAS framework, CDC school environment framework, and graziose socioecological framework with Canada’s National School Food Policy guiding principles and actionable objectives

Guiding Principle	Objectives^1^	INFORMAS Dimensions^2^ [25] (Physical, Economic, Socio-cultural, Policy)	CDC Components^3^ [29] (School meals, Competitive foods, Classroom celebrations, Access to drinking water, Staff role modelling, Food marketing, Healthy eating learning)	Graziose Factors^4^ [30] (School environment/policy, Cafeteria environment, Meal-specific, Item-specific, Individual)
Accessible Children and youth can participate in school food programs without stigma or barriers.	• Progressively expand toward universal access	Economic	School meals	Individual
	• Prioritize underserved communities	Economic	N/A	Individual
	• Reduce financial, physical, and social barriers to participation	Economic, Socio-cultural	School meals	School environment/policy
	• Strive for free meals at point of service to reduce stigma	Physical, Economic	School meals, Access to drinking water	School environment/policy
Health Promoting Food served is consistent with Canada’s Food Guide, and children and youth are supported in developing healthful food-related behaviors, knowledge, and skills.	• Align meals with Canada’s Food Guide and provincial/territorial nutrition standards	Physical, Policy	School meals	School environment/policy, Meal-specific
	• Foster positive eating environments	Physical	School meals, Competitive foods, Access to drinking water, Staff role modelling	School environment/policy, Cafeteria environment, Meal-specific, Item-specific
	• Protect against marketing of unhealthy foods	Physical, Economic	Food marketing	School environment/policy, Cafeteria environment/policy
	• Promote food literacy and nutrition skills	Socio-cultural	Healthy eating learning	School environment/policy
Inclusive Children and youth have access to culturally appropriate, relevant and inclusive SFPs that engage students and the broader community.	• Ensure programming is culturally relevant and responsive to diverse dietary needs	Physical, Socio-cultural	School meals	School environment/policy
	• Involve students, parents, and communities in program design	Socio-cultural	N/A	N/A
	• Leverage Indigenous leadership and perspectives	Socio-cultural	N/A	N/A
Flexible Food is locally sourced where possible and reflective of local and regional circumstances.	• Adapt programming to regional and community contexts	Economic	N/A	N/A
	• Support local decision-making and input	Policy	N/A	N/A
	• Incorporate student engagement in menu planning and food preparation	Socio-cultural	N/A	N/A
Sustainable Programs are designed to be environmentally sustainable, and adequately resourced.	• Strengthen and expand funding for long-term viability	Economic	N/A	N/A
	• Invest in infrastructure, staffing, and training	Physical, Economic	N/A	N/A
	• Encourage sustainable food procurement and practices to reduce food loss and waste	Policy	N/A	School environment/policy
Accountable Consistent and transparent monitoring and evaluation to ensure that programs are achieving policy objectives.	• Provide clear, transparent program information	Policy	School meals	School environment/policy
	• Ensure mechanisms for feedback from families and communities	Socio-cultural, Policy	N/A	N/A
	• Promote equitable monitoring and reporting to track progress	Policy	N/A	N/A

*Abbreviations:* *INFORMAS *International Network for Food and Obesity/non-communicable diseases research, monitoring and action support, *CDC* Centers for Disease Control and Prevention

^1^These objectives guided by the six key principles are listed and further described in Canada’s National School Food Policy [10]

^2^Dimensions of a school food environment. Descriptions of the dimensions have been paraphrased or adapted from a previous review by O’Halloran and colleagues [25]

^3^Components of a school food environment as outlined by the Centers for Disease Control and Prevention, USA [29]

^4^Factors that influence student nutrition behaviours as defined by Graziose [30]

#### Dimensions of school food environments

Information on school food environments captured by measurement tools was evaluated using Swinburn and colleagues’ food environment framework that adapted the widely accepted INFORMAS definition of the food environment [28]. Outcomes of measurement tools were categorized into the framework’s four food environment dimensions: (1) physical; (2) economic; (3) socio-cultural; and (4) policy. As the focus of the original framework was understanding the obesogenicity of food environments [28], we adapted this framework by expanding the definition of the socio-cultural environment to include the perceptions, attitudes, knowledge, and beliefs of students, school staff, and parents, as they relate to the school food environment (hereafter “INFORMAS dimensions”) ( Supplementary Material 2).

#### Centers for Disease Control and Prevention (CDC) – comprehensive framework for addressing the school nutrition environment and services

The CDC framework outlines seven components of the school food environment [29]: (1) School Meals; (2) Smart Snacks in School; (3) Classroom Celebrations, Events, and Non-food Rewards; (4) Access to Drinking Water; (5) Staff Role Modelling; (6) Food and Beverage Marketing; and (7) Healthy Eating Learning Opportunities. For the purposes of our review, we amended the component descriptions to be more generalizable and appropriate for the Canadian context. Descriptions can be found in Supplementary Material 2.

#### Socioecological framework of environmental influences on elementary students’ consumption of fruits and vegetables at school lunch

The socio-ecological framework presented by Graziose and Ang pieced together existing models to explore the overarching factors that influence students’ nutrition behaviors [30, 31]. In Graziose’s dissertation [30], the framework was further tailored to the school food environment by defining five factors: (1) school environment and policy factors; (2) cafeteria environment factors; (3) meal-specific factors; (4) item-specific factors; and (5) individual factors. We further amended the definition of “School Environment and Policy Factors” to include participation in any nutrition policy or program, not limited to the United States’ National School Lunch Program (NSLP). Definitions can be found in Supplementary Material 2.

#### Principles of Canada’s National School Food Policy

A sub analysis of Canadian studies was conducted to investigate whether existing measurement tools capture the six guiding principles outlined in Canada’s National School Food Policy that aims to support progress towards the policy’s vision [10]. The principles are:(1) Accessible; (2) Health promoting; (3) Inclusive; (4) Flexible; (5) Sustainable; and (6) Accountable. Descriptions for each guiding principle are presented in Table 1.

#### Quality assessment of measurement tools

The quality of measurement tools was assessed using criteria adapted by O’Halloran et al. from the INFORMAS quality assessment tool [25]. These criteria include (1) comprehensiveness (2), relevance (3), generalizability, and (4) feasibility. Each measurement tool was given a quality rating for each category (Low, Medium, or High), and then assigned an overall quality rating based on a combination of these ratings. Tools were rated High if they had mostly “High” ratings with no “Low,” Low if they had two or more “Low” ratings, and Medium if they fell between these two thresholds. Detailed criteria are available in Supplementary Material 3.

## Results

The systematic search yielded 101 articles that met the inclusion criteria and were included in this review [32–132] (Table 2). Articles were published between 2001 and 2024. Most studies used quantitative methods, while others used qualitative or mixed methods to measure the school food environment. Major reasons for exclusion were that studies (1) did not specify methods or tools used to assess school food environments, (2) focused on food environments outside of the specified school settings, and (3) measured pre/post-intervention related to school environments.

**Table 2:** Summary of the included studies featuring measurement tools capturing the dimensions of school food environments and the determinants of student eating behaviors [32–132]. Total *n* = 101

Author (reference)	Study Country	Objective of Study	Setting & Population	INFORMAS Dimensions	CDC Components	Graziose Factors	Tools Used	Outcomes Measured	Methodology	Overall Quality of Tool
Acciai et al., 2019 [32]	USA	To measure students’ exposure to school food and physical activity environment.	*n* = 141 primary & secondary public schools in New Jersey	Physical; Sociocultural; Policy	School meals; Smart snacks	School environment and policy; Meal-specific	Questionnaire	Healthy/unhealthy items available at school.	Quantitative	Medium
Alston et al., 2019 [33]	Australia	To determine the association between school food environments, dietary intake and BMI.	*n* = 3,496 students at 53 primary schools in Victoria	Physical; Sociocultural; Policy	School meals; Smart snacks; Healthy eating learning opportunities	School environment and policy; Cafeteria environment; Meal-specific; Individual	Dietary questionnaires; school audit.	Daily fruit and sugary drink consumption; presence of vegetable gardens, breakfast & healthy canteen programs; school socioeconomic status.	Quantitative	Medium
Andretti et al., 2021 [34]	Brazil	To assess how changes in cafeteria food and beverage quality affect subsequent (un)healthy purchases.	*n* = 20,233 students from 54 private schools in Brazil	Physical; Policy	School meals	School environment and policy; Meal-specific	Student transactions at school assessed by company database.	Daily purchases; nutritional value of food and beverages offered by school cafeteria.	Quantitative	Medium
Arcan et al., 2013 [35]	USA	To examine food practices and beliefs among students and teachers and determine impact of obesity intervention training.	*n* = 14 elementary school’s teachers on American Indian reservations	Physical; Sociocultural; Policy	Smart snacks; Staff role modeling	School environment and policy; Cafeteria environment; Individual	Two surveys.	Teacher classroom food-related practices; eating habits; teacher perceptions of schoolwide food practices and policies.	Quantitative	Low
Azaredo et al., 2016 [36]	Brazil	To evaluate the association between regular unhealthy food consumption and the school food environments.	*n* = 109,104 ninth grade students in public and private schools	Physical; Sociocultural; Policy	Smart snacks	School environment and policy; Meal-specific; Individual	Brazilian National Survey of School Health; questionnaires.	Sociodemographic data; unhealthy food intake; cafeteria menu items; food sold in nearby and on-site retailers.	Quantitative	Medium
Babashahi et al., 2021 [37]	Iran	To analyze the Healthy School Canteen policy and identify implementation challenges for improving the school food environment in Iran.	*n* = 64 primary schools in Tehran province	Physical; Economic; Sociocultural; Policy	School meals; Healthy eating learning opportunities	School environment; Individual	Semi-structured interviews, document review, direct observation; Food items data.	Food items in Tehran primary school canteens.	Mixed	High
Bardin et al., 2020 [38]	USA	To examine how school food environment healthfulness and NSLP lunch quality vary by student poverty levels and racial/ethnic composition.	*n* = 1,207 public, non-charter primary, middle, and high schools	Physical; Sociocultural; Policy	School meals; Smart snacks; Healthy eating learning opportunities	School environment and policy; Meal-specific; Item-specific; Individual	Checklists; menu audit.	Presence and sources of competitive foods; school food policies and programs; nutrition promotion activities; nutritional quality of NSLP lunches; sociodemographic data.	Quantitative	Medium
Belansky et al., 2010 [39]	USA	To assess the impact of the Local Wellness Policy in rural schools in Colorado.	*n* = 45 elementary schools in rural Colorado	Physical; Sociocultural; Policy	School meals; Smart snacks; Classroom celebrations; Access to drinking water	School environment and policy; Cafeteria environment; Meal-specific; Item-specific; Individual	Interviews with foodservice managers; self-report surveys.	Demographics; wellness policy; food offerings; lunch duration; serving methods; vending contents; recess timing; celebration foods; manager perceptions.	Mixed	Low
Betts et al., 2022 [40]	USA	To assess how school food availability relates to intake frequency and BMI, and the influence of neighborhood food outlets on these relationships.	*n* = 2,263 10th graders from 72 high schools in United States	Physical; Economic	School meals; Smart snacks	School environment; Cafeteria environment	Surveys.	Anthropometrics; demographics; school food availability; external food environment; neighborhood factors; student food intake frequency.	Quantitative	Low
Bhatt et al., 2019 [41]	India	To assess the consumption patterns of school canteen food among children in Mumbai.	*n* = 300 children, 10–12 y/o from schools in Mumbai	Physical; Economic; Sociocultural; Policy	School meals; Smart snacks	School environment and policy; Meal-specific; Item-specific; Individual	Questionnaire; food menu; interviews with canteen operators; researcher observation.	Food availability; accessibility; healthiness; canteen perceptions; guideline adherence; and school authority roles.	Mixed	Medium
Black et al., 2015 [42]	Canada	To assess the integration of healthy and sustainable food initiatives in elementary and secondary schools.	*n* = 32 elementary and secondary schools in Vancouver	Physical; Economic; Sociocultural; Policy	School meals; Smart snacks; Classroom celebrations; Healthy eating learning opportunities	School environment and policy; Cafeteria environment; Meal-specific; Item-specific	Field observations; interviews with stakeholders.	Eating spaces; food sales points; availability; presentation; healthy/sustainable eating policies; stakeholder insights.	Qualitative	High
Brennan et al., 2021 [43]	United Kingdom	To assess the intervention’s effect on children’s health-related quality of life, wellbeing, food knowledge, and dietary habits.	*n* = 903 students from 15 primary schools in Northern Ireland	Sociocultural; Policy	School meals; Smart snacks; Food and beverage marketing; Healthy eating learning opportunities	School environment and policy; Meal-specific; Individual	Randomized-controlled trial.	Dietary intake; emotional and behavioral wellbeing and health-related quality of life; cooking competence and food-related knowledge.	Quantitative	Medium
Briggs & Lake, 2011 [44]	United Kingdom	To evaluate and describe the home and school food environments of children.	*n* = 27 children, 8–10 y/o from a primary school in the suburb of Newcastle	Physical; Sociocultural	School meals; Access to drinking water	School environment and policy; Cafeteria environment	Photographs of meals; group interviews; informal observations; menu audit; discussion with school cook.	School meal preferences of students; food intake; menu content and availability.	Mixed	Low
Brouse, 2009 [45]	USA	To examine the perceptions of school food service directors on barriers/strategies for improving the school food environment and nutrition.	*n* = 508 food service directors in schools	Sociocultural	Healthy eating learning opportunities	School environment and policy	Self-report questionnaire.	Demographic; perceived barriers and strategies to improving healthy food choices.	Quantitative	Low
Bullock et al., 2010 [46]	USA	To collect and analyze data on competitive foods in schools, and evaluate adherence to California nutrition policies.	*n* = 21 schools in California	Physical	School meals	Meal-specific	Analysis of nutrition composition using software.	Nutrition composition of foods and beverages; percent adherence to the school nutrition policy.	Quantitative	Low
Burkhart et al., 2023 [47]	Pacific Islands region	To explore learnings associated within existing school food programs, and barriers to adopting the program.	*n* = 14 stakeholders from 11 countries in the Pacific island’s region, *n* = 2 representing regional organizations	Physical	School meals; Smart snacks	School environment and policy	Workshop sessions with stakeholders.	SFP policy presence; challenges (local food environments, alignment, advocacy, leadership, collaboration); integration opportunities.	Qualitative	Low
Callaghan et al., 2010 [48]	Canada	To evaluate the impact of a healthy vending machine pilot project.	*n* = 40 grade 9–11 students from 4 Ontario secondary schools	Physical; Policy	Smart snacks	School environment and policy	Focus groups; vending machine inventory.	How well healthier snacks sell; student perception of healthier snack options in vending machines.	Mixed	Low
Carter & Swinburn, 2004 [49]	New Zealand	To examine the impact of school food environments on student health and obesity.	*n* = 200 primary and intermediate school teachers; *n* = 26 secondary school teachers	Physical; Economic; Sociocultural; Policy	School meals; Smart snacks; Classroom celebrations; Staff role modeling; Healthy eating learning opportunities	School environment and policy; Meal-specific	Self-report questionnaire.	Facilities and food availability; menu prices; policies; nutrition prioritization; perceptions of food; quantity of menu items sold.	Quantitative	High
Chote et al., 2022 [50]	New Zealand	To measure the comprehensiveness and strength of school food policies.	*n* = 127 principal, senior management, health/nutrition teacher in Hawke’s Bay	Physical; Economic; Sociocultural	School meals; Food and beverage marketing; Healthy eating learning opportunities	School environment; Individual	Questionnaires answered by school staff.	Part A: Nutrition policies and programs; Part B: Food and beverage provision and sales (fundraising, celebrations, rewards), water fountains, sales methods, and types of food sold; Part C: School culture around healthy eating.	Mixed	Medium
Clarke et al., 2015 [51]	USA	To determine whether perceptions of home and school food environments are related to food and beverage intakes of postpartum teens.	*n* = 853 postpartum teens enrolled in a weight-loss study	Physical; Economic; Sociocultural; Policy	School meals; Smart snacks	School environment and policy; Meal-specific; Individual	Questionnaire; anthropometric measurements.	Perceived access of 4 groups of food items at home and school; dietary behaviors (using the SBFFQ).	Quantitative	Medium
Cullen et al., 2005 [52]	USA	To assess the impact of reducing sugar sweetened beverages and high-fat, salt, and sugar food portions on energy intake of students.	Middle school students from *n* = 23 schools	Physical; Policy	School meals	Meal-specific	Sales data; kilocalories of sold food products.	Snack bar sales; students’ energy intake.	Quantitative	Low
Cullen et al., 2006 [53]	USA	To assess the impact of changes in school food policy on student lunch consumption in middle schools.	*n* = 2,790 grade 6–8 students from three middle schools	Physical; Policy	School meals	School environment and policy; Meal-specific; Individual	Food records; point of service machine electronic data files.	Amount and source of food and beverages; snack bar sales.	Quantitative	Low
Cullen et al., 2007 [54]	USA	To examine the feasibility of instituting school environmental changes during a 6-week pilot in school foodservice programs.	Students and staff at *n* = 6 middle schools	Physical; Policy	School meals	School environment and policy	Questionnaires; daily food production and sales record.	Feedback from students and staff on policy implementation; adherence to 13 food service goals.	Mixed	Low
Czarniecka-Skubina et al., 2023 [55]	Poland	To identify food and meal organizations in primary schools through feedback from school staff and catering representatives.	*n* = 48 participants (24 headmasters/canteen workers, 24 catering reps) from 24 primary schools	Physical; Sociocultural; Policy	School meals; Access to drinking water	School environment and policy	In-depth interviews.	Meal organizations; school shop/vending operations; preferences & satisfaction; food waste; ideal system; COVID-19 adjustments; issues; improvement prospects.	Qualitative	Low
D’Souza et al., 2022 [56]	New Zealand	To evaluate the healthiness of New Zealand school food environments.	*n* = 819 schools	Physical; Economic; Policy	School meals	School environment and policy	Questionnaire; canteen menu analysis.	School nutrition policies and canteen menus.	Quantitative	Medium
de Assis Correia et al., 2022 [57]	Brazil	To assess association between schools’ internal environment factors and its surroundings with obesity in adolescents.	*n* = 2,530 adolescents in public and private schools in Belo Horizonte City	Physical	School meals; Smart snacks	School environment; Individual	Anthropometric measurements; questionnaire.	Obesity; school managerial dependence type; number of operational drinking fountains; school sports environment; food sales environment around the school.	Quantitative	Low
Evans et al., 2021 [58]	USA	To investigate stakeholders’ perspectives on the school food environment since the school nutrition reform.	*n* = 42 faculty and/or staff from 14 public middle schools	Physical; Economic; Sociocultural; Policy	School meals; Smart snacks; Classroom celebrations	School environment and policy	Interviews with key stakeholders.	Stakeholder views on nutrition reform: awareness, attitudes, benefits, barriers, food quality, taste, student behaviors, and policy improvement recommendations.	Qualitative	High
Fitzpatrick et al., 2017 [59]	Canada	To examine the relationship between school indoor dietary environment, the surrounding school neighborhoods and adiposity in children.	*n* = 431 children, 8–10 y/o who are at risk of obesity from 246 primary schools in Montreal	Physical; Sociocultural	School meals; Smart snacks	School environment and policy; Meal-specific; Individual	Survey; Geographic Information System; census data for neighborhood deprivation; adiposity measures.	Cafeteria menu; healthy food for sale; school neighborhood socioeconomic status; anthropometric measurements; family socioeconomic status.	Quantitative	Low
Foo et al., 2021 [60]	Malaysia	To assess the type and quality of food and beverages sold in canteens in public schools.	*n* = 140 public schools (97 primary and 43 secondary) in Kelantan	Physical; Economic	School meals	Meal-specific; Item-specific	Direct observations in school canteens.	Food and beverage ingredients; size of food and beverage served; total calories, fat, sugar, and salt.	Quantitative	Medium
Gangemi et al., 2020 [61]	USA	To assess adolescents’ views on their food environments through photographs and corresponding interviews.	*n* = 20 adolescents from a high school in Philadelphia	Physical; Sociocultural; Policy	School meals; Smart snacks	Meal-specific	Photo-elicitation; non-structured interviews.	Healthfulness of school vending machines; lunch choices (home vs. school); factors influencing students’ food choices; critiques of school food environments.	Qualitative	Low
Gebremariam et al., 2012 [62]	Norway	To investigate the food environment in elementary schools and assess dietary behaviors children.	11 y/o students and principals and teachers at 35 elementary schools	Physical; Sociocultural; Policy	School meals; Staff role modeling; Food and beverage marketing	School environment and policy; Meal-specific; Individual	Frequency questionnaire for dietary assessment; questionnaires.	Dietary behaviors; demographic analysis; school-level factors (responsibility, food/nutrition prioritization, nearby food sales, nutrition guidelines).	Quantitative	Medium
Godin et al., 2019 [63]	Canada	To examine associations between Canadian adolescents’ sugar-sweetened beverage consumption and school food environment characteristics.	*n* = 41,829 students at 89 secondary schools in Ontario and Alberta	Physical; Sociocultural	Smart snacks; Access to drinking water	School environment and policy; Cafeteria environment; Meal-specific; Individual	COMPASS student questionnaire; COMPASS School Environment Application (Co-SEA) and Desktop Mapping Technologies Inc.	Student SSB intake; SSB availability; in-school water fountain access; food outlets within a 1 km radius of schools.	Quantitative	Medium
Goldberg et al., 2009 [64]	USA	To describe the improvement of nutrient quality of school meals and associated barriers/facilitators.	Students, food service staff, parents, teachers and school leaders at elementary school	Physical; Sociocultural; Policy	School meals; Healthy eating learning opportunities	School environment and policy; Meal-specific	Focus groups or interviews with school employees, students, parents, guardians.	Dietary behaviors; menu changes; staff training; awareness, attitudes, and behavior changes in the school food environment.	Qualitative	Low
Gonçalves et al., 2019 [65]	Brazil	To characterize the food environment and to identify factors associated with hypertension and obesity.	*n* = 73,399 students in secondary schools, 12–17 y/o	Physical; Sociocultural; Policy	School meals; Food and beverage marketing	School environment and policy; Meal-specific; Individual	Self-administered survey; interviews with school principals; observations on school food environment.	Anthropometrics; school location/type; school meals and competitive foods; nearby food retailers; food advertisements.	Mixed	Medium
Gordon et al., 2009 [66]	USA	To examine the role of low-nutrient energy dense foods in children’s diets and the role of school meal environments in shaping dietary behaviors.	*n* = 2,314 grades 1–12 students, principals, foodservice directors at 287 schools	Physical; Sociocultural; Policy	School meals; Smart snacks; Classroom celebrations	School environment and policy; Meal-specific; Item-specific; Individual	Menu analysis; questionnaires; direct observation; 24-hour dietary recall.	Availability and consumption of competitive foods; their energy contribution; school food environment; policies; lunch characteristics; dietary behavior; food opinions.	Mixed	Medium
Gosliner et al., 2011 [67]	USA	To elucidate student perception of school food environments and assess correlations between perceptions, purchasing, and consumption at school.	*n* = 5,365 students at 19 schools in multi-ethnic, low-income California neighbourhood	Sociocultural	School meals	School environment and policy; Meal-specific	Knowledge, attitudes, and behavior assessment; questionnaire and intakes assessment.	Food intakes and KAB regarding school food environment	Quantitative	Low
Graves et al., 2008 [68]	USA	To assess the school breakfast environment in rural Appalachian schools.	Fourth & fifth grade students and food service managers at 4 rural schools	Physical; Policy	School meals	Meal-specific; Item-specific	Menu documentation; interviews.	School food environment and nutrition composition	Mixed	Low
Green et al., 2018 [69]	USA	To evaluate the impacts of the Philadelphia Campaign for Healthier Schools.	*n* = 100 schools (kindergarten – grade 8) in Philadelphia	Physical; Sociocultural; Policy	Smart snacks; Classroom celebrations; Food and beverage marketing; Healthy eating learning opportunities	School environment and policy; Meal-specific	Interviews and surveys with school staff before and 1 year into the campaign.	Availability of classroom foods; corner store foods; vending machine items; after-school foods; school-sold foods; corporate marketing; and health promotion activities at baseline and follow-up.	Qualitative	Medium
Gross et al., 2019 [70]	USA	To examine how the physical cafeteria environment contributes to school food consumption.	Students, 6–8 y/o at 10 New York City public elementary schools	Physical	School meals	Meal-specific and Item-specific	Before and after lunch tray photos; checklist for cafeteria’s physical environment.	FV and whole-grain consumption; physical environment of cafeteria	Quantitative	Low
Gustafson et al., 2017 [71]	USA	To examine the effects of food purchasing patterns and the home, school, and consumer food environments on dietary intake among rural adolescents.	*n* = 432 high school students from 8 rural high schools in Kentucky and North Carolina	Physical; Economic; Sociocultural; Policy	Smart snacks; Food and beverage marketing; Healthy eating learning opportunities	School environment and policy; Cafeteria environment; Meal-specific; Individual	Surveys; school and consumer food environment audits.	Home and school food environments; consumer food environment; food purchasing patterns; dietary intake of fruits, vegetables, and added sugars	Quantitative	High
Hadi et al., 2021 [72]	Indonesia	To describe the characteristics of school canteens in Indonesia and examine impact of canteen management on food quality.	*n* = 152 primary schools (private, public, religious)	Physical; Sociocultural	School meals; Smart snacks; Access to drinking water	School environment; Individual; Item-specific	Survey administered to principals; Interviews with heads of elementary schools via Zoom.	Healthy Canteen Score: canteen presence, nutrition curriculum, food storage, food availability, restrictions, sales bans, outside food limits, and oversight.	Mixed	Medium
Hermans et al., 2020 [73]	Netherlands	To examine adolescents’ food purchasing patterns in and around school and its relationship with perceived maternal relationship support and monitoring.	*n* = 726 adolescents and *n* = 713 mothers at primary schools	Physical; Sociocultural	Smart snacks	Meal-specific; Individual	Food Frequency Questionnaire; self-report questionnaires.	Adolescents’ frequency of bringing and purchasing foods; perceived maternal relationship support and monitoring	Quantitative	Low
Huang et al., 2017 [74]	China	To evaluate the intake of food and nutrients among students and provide recommendations for new school lunch standards.	Primary, middle, and high school students in Shanghai	Physical; Sociocultural	School meals	Meal-specific; Individual	Menu surveys analyzed by a nutrient analysis software; plate waste measures; questionnaire.	Menu evaluation; food intake frequency; knowledge and attitudes about food, nutrients, and opinions on food served	Quantitative	High
Hugues et al., 2021 [75]	Mexico	To describe the implementation of, and compliance with, the 2014 version of the AGREEMENT in a representative sample of elementary schools.	*n* = 119 schools, median number of students per school was 237	Physical; Economic; Sociocultural	School meals	School environment and policy; Cafeteria environment	Healthy Canteen Score.	Implementation and compliance with Agreement; barriers and facilitators; water fountain availability/quality; processed food/beverage advertising.	Mixed	Medium
Iyassu et al., 2024 [76]	Ethiopia	To assess factors that influence dietary behaviors of adolescents in urban Ethiopia.	*n* = 432 students, 15–19 y/o and *n* = 36 school principals from 12 secondary schools in three cities of Ethiopia	Physical; Sociocultural; Policy	School meals; Classroom celebrations rewards	School environment and policy; Item-specific; Individual	Focus groups with photovoice; interviews.	Principal and student perspectives on dietary behaviors; school food policies; barriers to healthy eating.	Qualitative	Low
Jensen et al., 2021 [77]	Costa Rica	To assess the extent to which mandatory school food environment guidelines were implemented in high schools and the perspectives of key policy actors.	*n* = 22 participants (principles and kiosk concessionaires) in San José	Physical; Economic; Policy	School meals; Smart snacks	School environment and policy; Item-specific	Two semi-structured interviews; checklist; site observations.	Opinions on student health; food availability; recent changes and reasons; assessment of on-site prepared foods and beverages.	Qualitative	Medium
Jones et al., 2011 [78]	USA	To test the effectiveness of using a Positive Youth Development framework to train youth leaders in rural schools to plan, implement, and evaluate school nutrition environment interventions.	*n* = 350 grade 4–5 students from five rural East Tennessee County schools	Physical; Sociocultural	School meals; Healthy eating learning opportunities	School environment and policy; Individual	24-hour dietary recall and surveys.	Intake; knowledge, attitudes, and behaviors on food environment.	Quantitative	Medium
Kilandeka et al., 2024 [79]	Dodoma	To examine the school food environment among primary school children and its relationship with their eating behaviors.	*n* = 248 primary school children in urban and rural areas	Physical	School meals; Smart snacks	School environment; Individual	Questionnaire.	A: rapport; B: socio-economic data; C: eating behaviors.	Quantitative	Low
Krukowski et al., 2011 [80]	USA	To develop a comprehensive, standardized, and validated measurement of school cafeterias.	*n* = 125 public schools	Physical	School meals	Cafeteria environment; Meal-specific	School cafeterias measured using a scoring system.	School meals and lunches (i.e. foods available); demographics.	Quantitative	Medium
Kubik et al., 2010 [81]	USA	To examine if nutrition policies on junk food in school vending machines and stores were associated with lower availability of junk food.	*n* = 538 public school districts across all 50 states	Physical; Policy	School meals; Smart snacks; Classroom celebrations; Staff role modeling	School environment and policy; Meal-specific	Telephone interviews; self-administered mail questionnaires.	State/district requirements on junk foods; student access to food or beverages from vending machines, school stores, canteens, or snack bars.	Mixed	High
Lambert et al., 2016 [82]	USA	To investigate teachers’ behavior towards using foods with low nutritional value in the classroom.	*n* = 280 teachers from 10 public elementary schools in 3 northern Mississippi school districts	Physical; Sociocultural; Policy	Classroom celebrations; Staff role modeling	School environment and policy; Cafeteria environment; Meal-specific	Surveys.	Teacher allowances for low-nutritional-value foods in the classroom; frequency of rewards; teacher attitudes.	Quantitative	Medium
Leite et al., 2021 [83]	Brazil	To investigate the association between the presence of canteens and availability of ultra-processed foods with the consumption of these foods, inside schools.	*n* = 2,680 9th grade students from 119 schools Sao Paulo	Physical; Economic; Sociocultural	School meals; Smart snacks; Food and beverage marketing	School environment; Individual	Questionnaires; researcher observation.	Commercial canteens; ultra-processed food sales; unhealthy food markers; lack of cafeterias; alternative food outlets; nearby street vendors; food ads inside schools.	Quantitative	Medium
Lien et al., 2014 [84]	7 European countries	To describe practices related to the school food environment in 7 European countries.	10–12 y/o and school management	Physical; Sociocultural; Policy	School meals; Smart snacks; Classroom celebrations; Access to drinking water; Staff role modeling; Healthy eating learning opportunities	School environment and policy; Cafeteria environment; Meal-specific; Individual	Questionnaires; anthropometric measurement; interviews; school environment audit instrument.	Student demographics; food availability; school nutrition policies; staff roles in nutrition modeling and teaching; and student soft drink consumption frequency.	Mixed	High
Longacre et al., 2014 [85]	USA	To examine whether school food reduces household income-related disparities in adolescents’ frequency of fruit and vegetable intake.	*n* = 1,542 grade 4–6 students at 26 public schools in New Hampshire and Vermont	Physical; Economic; Sociocultural	N/A	School environment and policy; Meal-specific; Individual	Longitudinal telephone survey with students and parents.	Adolescent frequency of fruit and vegetable intake.	Qualitative	Medium
Lytle et al., 2006 [86]	USA	To examine the effects of an intervention for increasing the availability of fruits, vegetables, and lower fat foods in homes and schools.	*n* = 3,600 middle school students at 16 schools	Physical; Policy	School meals; Healthy eating learning opportunities	School environment and policy; Meal-specific; Individual	Survey; food availability data; observations of school meals and number of students served.	Home and school food environments (i.e., foods available).	Quantitative	Medium
Martinelli et al., 2021 [87]	USA	To compare the healthfulness of foods offered in schools with parental perception of school meals.	*n* = 1,201 students from public schools in Camden, New Brunswick, Newark and Trenton	Physical; Sociocultural	School meals; Smart snacks	School environment	Computer-assisted phone interviews and surveys.	Demographics; relationship between school food environment and perceptions.	Quantitative	Medium
Martinez-Ospina et al., 2019 [88]	Colombia	To examine the school food environment and the relationship of consumption and physical activity with overweight/obesity.	7–14 y/o students at 10 elementary and secondary education schools in Bogota	Physical; Sociocultural	School meals; Smart snacks; Access to drinking water	School environment and policy; Meal-specific; Individual	Surveys; field assessment; anthropometric measurements	Food consumption; physical activity; sleep; access to stores; health; access to healthy foods/water; health knowledge; anthropometric measures.	Quantitative	Medium
Masse et al., 2013 [89]	Canada	To determine whether the availability of certain foods was associated with school environment policies.	Public school principals at elementary and middle schools in British Columbia	Physical; Sociocultural; Policy	School meals; Smart snacks; Classroom celebrations; Staff role modeling; Food and beverage marketing; Healthy eating learning opportunities	School environment and policy; Meal-specific	Survey.	Nutrition policies and guidelines; qualifications of food personnel; nutrition education at schools.	Quantitative	Low
Mauti et al., 2021 [90]	Burkina Faso	To better understand the food and health environment across schools.	*n* = 19 school administrators; *n* = 18 food vendors; *n* = 1,059 in-school adolescents from 22 schools	Physical; Economic; Sociocultural; Policy	School meals; Smart snacks; Access to drinking water.	School environment and policy; Meal-specific; Item-specific; Individual	Structured questionnaires administered by field workers.	Health policies; nutrition; WASH; physical activity; healthcare access; food sales; hygiene; preferences; security; peer influence; mental health.	Quantitative	High
McIsaac et al., 2015 [91]	Canada	To evaluate policy adherence of school lunch menus in Nova Scotia.	*n* = 110 public elementary schools in Nova Scotia	Physical; Sociocultural; Policy	School meals; Smart snacks	School environment and policy; Meal-specific	Menu analysis	Menu items and nutrition category (i.e., healthy versus unhealthy).	Quantitative	Low
Moore et al., 2023 [92]	Ireland	To develop and test an instrument assessing the school food environment, and provide recommendations.	*n* = 18 schools in urban and rural geographical regions of Northern Ireland of varying socio-economic status	Physical; Sociocultural; Policy	School Meals; Staff Role Modelling	School Environment and Policy; Meal-specific	Observation; intake monitoring; questionnaire.	Physical setting; food policies; food provision; parental engagement in school food.	Quantitative	High
Neumark-Sztainer et al., 2005 [93]	USA	To describe school lunch practices and vending machine purchases among high school students, and associations between their eating patterns and school food environment.	*n* = 1,088 high school students from 20 high schools in metropolitan areas in Minnesota	Physical; Policy	School meals; Smart snacks	School environment and policy; Meal-specific; Item-specific	Self-administered surveys; researcher observation.	Number and hours of operation of vending machines; existence of nutrition policies; how often students bought lunch from school versus brought lunch from home.	Quantitative	Medium
Newton et al., 2011 [94]	USA	To evaluate the implementation of a multicomponent childhood obesity prevention intervention in rural schools.	*n* = 232 classroom teachers; *n* = 53 physical education teachers; *n* = 33 food service managers; *n* = 9 trained observers at 17 rural Louisiana schools	Physical; Sociocultural; Policy	School meals; Healthy eating learning opportunities	School environment and policy; Cafeteria environment; Individual	Questionnaires and observations.	School cafeteria practices; health guidelines and recommendations; physical activity education; school cafeteria environment.	Mixed	Medium
Nollen, 2007 [95]	USA	To examine the perceptions of school staff on school food environment in relation to obesity and legislation.	*n* = 8 high school principals, *n* = 7 dietitians/food service managers	Sociocultural; Policy	N/A	School environment and policy; Individual	Semi-structured interviews.	Perceptions of student health; obesity; school nutrition policies; food offerings; barriers; student demographics.	Qualitative	Low
Noor et al., 2024 [96]	Sub-Saharan Africa region	To understand the school food environment through school administrators, food vendors, and adolescents.	*n* = 4,999 adolescents from 79 primary schools (22 in Burkina Faso, 20 in Ethiopia, 5 in South Africa, 11 in Sudan, and 21 in Tanzania)	Physical; Economic	School meals; Smart snacks; Food and beverage marketing	School Environment and Policy; Cafeteria Environment; Meal-specific	Structured questionnaires administered by field workers; surveys.	Availability of health policies, guidelines, and curricula; health, nutrition, and WASH services; food items from vendors in and around schools; school food environment and eating habits.	Quantitative	High
O’Donnell et al., 2022 [97]	USA	To assess facilitators and barriers to participating in the school meal program among adolescents of color.	*n* = 47 students; *n* = 24 caregivers across 4 public schools in the USA	Physical; Policy	School meals; Smart snacks; Healthy eating learning opportunities	School Environment and Policy; Cafeteria Environment; Meal-specific	Focus groups; key informant interviews.	Demographics, socioeconomic status; school meal participation; health habits; meal participation predictors; nutrition knowledge; communication preferences.	Qualitative	Medium
Ohri-Vachaspati et al., 2023 [98]	USA	To assess the association between school and community food environments and the prevalence of obesity.	106 public schools located in Camden, New Brunswick, Newark, and Trenton	Physical; Economic; Policy	Smart snacks	Meal-specific; Individual	Survey; systematic classification protocol.	Healthfulness of school lunches; competitive foods; community food environment (food outlets, NSLP, à la carte, and vending options).	Quantitative	Medium
Okamura et al., 2022 [99]	Brazil	To investigate the role of school feeding in insulin resistance.	*n* = 27,990 adolescents aged 12 to 17 from public and private schools	Physical; Economic	Smart snacks	School Environment and Policy; Meal-specific	Cross-sectional national data based on questionnaires or school observations.	School location area; sale of food at school; presence of vending machines; presence of advertising of industrialized foods; sale of food in the vicinity of the school.	Quantitative	High
Park et al., 2017 [100]	South Korea	To create and pilot test a school nutrition environment index.	7 elementary and 8 middle schools in Seoul	Physical; Economic; Sociocultural; Policy	School meals; Smart snacks; Staff role modeling; Healthy eating learning opportunities	School environment and policy; Meal-specific	Questionnaire for school dietitians; lunch menu analysis.	Resource availability; education and programs; dietitians’ perceptions and characteristics; school lunch menu.	Quantitative	High
Pehlke et al., 2016 [101]	Guatemala	To assess privately owned casitas (kiosks) in Guatemalan schools.	*n* = 4 school principals; *n* = 4 kiosk vendors; *n* = 48 children, 7–12 y/o at 4 schools in low income, peri-urban municipalities near Guatemala City	Physical; Economic; Sociocultural; Policy	Smart snacks	School environment and policy; Meal-specific	In-depth interviews; focus groups; direct observations.	Student preferences; food availability; staff perceptions of nutrition barriers (pricing, time, space); policy regulations/enforcement.	Qualitative	Low
Pillay et al., 2024 [102]	New Zealand	To assess primary school canteen food menus against the newly implemented Ministry of Health ‘Food and Drink Guidance for Schools’.	*n* = 133 primary school canteens	Physical; Sociocultural	School meals; Smart snacks; Access to Drinking Water; Healthy eating learning opportunities	School Environment and Policy; Individual	Questionnaire; menu analysis.	Healthiness of school food menus.	Mixed	Medium
Prelip et al., 2012 [103]	USA	To evaluate the impact of a multi-component school-based nutrition education program on students’ knowledge, attitude, and behaviors.	*n* = 339 grade 3–5 students at 6 elementary schools in low-income areas	Sociocultural; Policy	Staff role modeling; Healthy eating learning opportunities	School environment and policy; Meal-specific; Individual	Baseline and post-intervention questionnaires.	Fruit and vegetable (FV) consumption; knowledge, attitudes, and behaviors about food groups; parent and teacher influence on FV consumption; demographics.	Quantitative	Medium
Rathi et al., 2017 [104]	India	To describe the opinions of adolescents, parents, educators, and principals on food environment and canteen policies.	*n* = 15 adolescents; *n* = 15 parents; *n* = 12 teachers; *n* = 10 principals at 10 private secondary schools in Kolkata	Physical; Sociocultural; Policy	School meals; Smart snacks; Healthy eating learning opportunities	School environment and policy; Meal-specific; Individual	Semi-structured interviews.	Interviewee’s opinion on school food environment and whether it promotes healthy eating.	Qualitative	Medium
Richardson et al., 2020 [105]	USA	To assess the school food environments of children from military families in the USA.	*n* = 815 children, 12–13 y/o from US military families	Physical; Economic	Smart snacks	Meal-specific; Individual	Online surveys; anthropometric measurements.	Food intake (fruit, vegetables, soft drinks, sweets); purchasing behaviors; food availability; home environment; and demographics.	Quantitative	Medium
Rocha et al., 2021 [106]	Brazil	To examine the association between the school food environment and sugar-sweetened beverage consumption among adolescents.	*n* = 71,549 adolescents, 12–17 y/o; *n* = 1,247 schools in Brazilian cities with a population of >100,000	Physical; Economic	Smart snacks; Access to Drinking Water; Food and beverage marketing	School Environment and Policy; Meal-specific	24-h recall, systematic social observation using a questionnaire.	SSB consumption (mL/day); number of drinking fountains per 100 students; soft drink sales; soft drink advertising; presence of street vendors.	Quantitative	High
Rollings et al., 2018 [107]	USA	To quantify associations between elementary school cafeteria environments and students’ selection and consumption of fruits and vegetables.	*n* = 3,187 elementary school students at 50 schools in New York, Iowa, Arkansas, and Washington	Physical	Smart snacks; Food and beverage marketing	Cafeteria environment; Meal-specific; Item-specific; Individual	US census data: school tray photographs before and after eating analyzed by Digital Food Image Analysis software.	Demographics; room; table/display; plate; food scales; fruits and vegetables served and consumed.	Quantitative	Low
Schneider et al., 2009 [108]	USA	To describe the design and methods of evaluating the HEALTHY study.	Students and staff at middle schools	Physical; Policy	School meals	School environment and policy	Direct observations; interviews and focus groups.	Fidelity (intervention delivery); dose (amount); reach (recipient proportion); facilitators/barriers; participant feedback.	Mixed	Low
Schwartz et al., 2020 [109]	USA	To assess whether strong state-level policies are associated with lower student BMI-for-age percentiles.	*n* = 1,625 students in 284 schools in 30 states and the District of Columbia	Physical; Sociocultural; Policy	School meals	School Environment and Policy; Meal-specific; Individual	Nationally representative data of students enrolled in public, non-charter schools that participated in the National School Lunch Program.	Availability of competitive foods in vending machines, school stores, and à la carte; state laws; free/reduced lunch eligibility.	Mixed	Low
Stephenson et al., 2013 [110]	Canada	To evaluate of the potential impact of the Healthy Eating Guidelines Initiative on the school food environment and students’ self-reported diets.	*n* = 8,428 grades 7–12 students from 22 schools	Physical; Sociocultural; Policy	School meals; Smart snacks	School environment and policy; Cafeteria environment; Meal-specific	Interviews with school representatives; inspection list assessment; student questionnaire.	Type of food service available (full service, vending machine only, no service); student self-reported dietary behaviors during preceding 24 h.	Mixed	Medium
Steyn et al., 2015 [111]	South Africa	To assess the effectiveness of a primary school nutrition intervention on the dietary quality of children from low-income backgrounds.	*n* = 998 fourth grade students from 16 schools from low-income districts in Western Cape Province	Physical; Sociocultural; Policy	Smart snacks	School environment and policy; Meal-specific; Individual	Three wave surveys [2009–2011]; 24-hour recall.	Consumption of food and drinks by participating students over a 24-hour period; percent of participants who consumed foods high in fat and/or sugar.	Quantitative	Low
Taber et al., 2015 [112]	USA	To examine whether state competitive food laws reduce disparities in compliance across income levels.	5th and 8th grade students from 2,850 schools	Physical; Policy	Smart snacks	School environment and policy; Meal-specific	Questionnaire completed by school administrators; state codified laws extracted from secondary dataset.	Food and beverage items sold in school; state competitive food laws.	Quantitative	Medium
Teo et al., 2019 [113]	Malaysia	To evaluate the effectiveness of the School Nutrition Program.	7–11 y/o children from six selected schools in Batu Pahat District, Johor State, Malaysia	Physical; Sociocultural	School meals	Individual	Questionnaires; self-administered 7-day recall; Pediatric Quality of Life Inventory (five-point Likert scale)	Anthropometrics; nutrition knowledge, attitudes, behaviors; eating habits; physical activity; psychological distress; cognitive performance; quality of life in children.	Quantitative	Medium
Terry-McElrath et al., 2009 [114]	USA	To identify trends in foods available in schools and associations with intakes and BMI-related outcomes.	*n* = 78,442 students in 684 secondary schools and food service managers	Physical; Policy	School meals; Smart snacks	School environment and policy; Meal-specific; Individual	Questionnaires.	School food environment policies; self-reported anthropometrics (height and weight); self-reported student food consumption.	Quantitative	Medium
Thompson et al., 2010 [115]	USA	To evaluate the relationship between school vending machine purchasing behavior, access, and individual-level dietary characteristics.	*n* = 1,695 children and adolescents	Physical	Smart snacks	Meal-specific; Individual	Secondary analysis of a consumer mail panel survey (Youth Styles 2005 survey)	Vending machine purchasing behavior and access; dietary intake; and self-reported demographic data.	Quantitative	Medium
Trubswasser et al., 2022 [116]	Ethiopia	To assess whether food environments in and around schools influence dietary diversity, quality, BMI status or perceptions of adolescents.	*n* = 217 high school students in Addis Ababa, Ethiopia.	Physical; Economic; Sociocultural; Policy	School meals; Smart snacks; Access to Drinking Water;	School Environment and Policy; Meal-specific; Item-specific; Individual	Interviews; dietary intake assessments, and anthropometric measurements.	Socio-economic factors; purchasing behavior; food environment perceptions; dietary intake; anthropometrics; food assessment.	Mixed	High
Tsai et al., 2021 [117]	USA	To characterize school nutrition environments by identifying underlying factors and their relationships with child anthropometric and dietary outcomes.	Children from 386 schools in South, West, Midwest, and Northeast of the United States	Physical; Sociocultural; Policy	Access to Drinking Water; Food and beverage marketing; Healthy eating learning opportunities	School Environment and Policy; Meal-specific; Item-specific; Individual	Observations, surveys.	Nutrition education; food options; wellness policies; dining environment; unhealthy food restriction; nutrition programs.	Quantitative	High
Turner et al., 2012 [118]	USA	To examine changes in school food environment post-implementation of a wellness policy.	Principals and food service managers at 1,803 schools	Physical; Sociocultural; Policy	School meals; Smart snacks; Classroom celebrations; Healthy eating learning opportunities	School environment and policy; Cafeteria environment	16-item questionnaire.	Competitive foods; school lunches; kitchen facilities; participation in nutrition program; nutrition education; nutrition of staff.	Quantitative	Medium
van der Horst et al., 2008 [119]	Netherlands	To examine the association between availability of canteen food and drinks, the presence of food stores around school, and attitudes, behaviors, and knowledge.	*n* = 1,293 adolescents, 12–15 y/o from 17 secondary schools in Rotterdam	Physical; Sociocultural	School meals; Smart snacks	Meal-specific; Individual	Questionnaires and observations.	Cognitive factors (attitudes, parental norms, modeling, perceived control); snack and soft drink availability; school and local food environment; demographics.	Mixed	Medium
Vilela et al., 2023 [120]	Brazil	To evaluate the economic and financial aspects of different models of companies in the school canteen sector.	*n* = 6 outsourced companies responsible for 36 private school canteens.	Physical; Sociocultural	School meals; Smart snacks	School Environment and Policy; Meal-specific	Online questionnaire self-completed by canteen managers.	Food items sold in traditional vs. healthy canteens; nutritional content; student servings; costs; profit margins; investment recovery; profitability.	Mixed	Medium
Volpe et al., 2013 [121]	USA	To develop and test a school-based intervention aimed at reducing modifiable risk factors for type 2 diabetes in middle schools.	*n* = 4603 students from 42 middle schools across the USA	Physical; Economic; Sociocultural; Policy	School meals; Healthy eating learning opportunities	School environment and policy; Meal-specific; Item-specific	Observations; structured interviews; cafeteria menu analysis.	Nutrition goals; nutrition education and strategies; barriers and facilitators for a healthy food environment.	Mixed	Low
Weigel et al., 2022 [122]	Ecuador	To examine the association of school food sources with healthy and unhealthy food intake and BMI in students.	*n* = 12,632 primary, and *n* = 6,617 secondary students in Ecuadorian provinces	Sociocultural; Policy	School meals; Healthy eating learning opportunities	School Environment and Policy; Meal-specific; Individual	Questionnaire; food frequency questionnaire.	School bar presence; “red traffic light” foods availability; child intake of six food/beverage groups over a week; BMI from height and weight.	Quantitative	Medium
Whatley Blum et al., 2007 [123]	USA	To examine the impact of implementing guidelines in competitive food offerings.	*n* = 7 high schools in Maine	Physical	Smart snacks	Meal-specific; Item-specific	Observations; availability and nutrition profiles of foods in vending machines; portion size records.	Vending machines; competitive foods; nutrition profiles; barriers to low-fat; low-sugar; portion-controlled guidelines in schools.	Mixed	Low
Whatley Blum et al., 2011 [124]	USA	To assess the effect of nutrition policy on the food environment of public schools.	Food service directors at 89 high schools	Policy	School meals	School environment and policy	Observations; Survey.	Types of foods available; program evaluation.	Quantitative	Low
Wijnhoven et al., 2014 [125]	12 European countries	To assess differences in school nutrition environment and BMI in primary schools between and within 12 European countries.	Primary school students at 1,831 schools in 2007/2008 and 2,045 schools in 2009/2010	Physical; Sociocultural; Policy	N/A	School environment and policy; Meal-specific; Individual	Questionnaire completed by school personnel; anthropometric measures.	Physical education frequency; playground access; on-site food/beverage availability; healthy lifestyle initiatives; anthropometric measures.	Quantitative	Medium
Wolfenden et al., 2019 [126]	Australia	To assess whether improvements in primary school implementation of a mandatory state-wide healthy canteen policy were sustained.	*n* = 35 primary in the Hunter Region of New South Wales	Physical; Sociocultural; Policy	School meals; Smart snacks	School environment and policy; Meal-specific	Menu assessment using a quick menu audit tool.	Canteen menu compliance with state policies; proportion of schools without restricted item and those with >50% healthy menu options.	Quantitative	Medium
Woodward-Lopez et al., 2010 [127]	USA	To assess the impact of nutrition standards on school meals in California.	Students, food service directors, principals, and wellness team members at schools in California	Physical; Sociocultural; Policy	School meals; Smart snacks	School environment and policy; Meal-specific; Individual	Surveys; sales data.	Student food intake; purchases; attitudes; perceptions; sales data; factors influencing nutrition standards and school wellness policy implementation.	Quantitative	Medium
Yang & Kim, 2020 [128]	South Korea	To examine how household and school fruit environments influence fruit intake and overweight risk in low-income children and adolescents.	*n* = 3,148 students who were beneficiaries of Seoul’s Community Childcare Centre	Physical; Sociocultural	School meals	Meal-specific; Individual	Fruit frequency questionnaires.	Anthropometric measures; fruit intake at home and school; habit of frequently eating fruit among parents and friends.	Quantitative	Medium
Yazdi-Feyzabadi et al., 2018 [129]	Iran	To examine the role of Iranian Health Promoting Schools in improving the school food environment and snacking behaviors among adolescents.	*n* = 1,320 eight grade students from 40 middle schools in Kerman	Physical; Sociocultural; Policy	Smart snacks; Healthy eating learning opportunities	School environment and policy; Meal-specific; Individual	55-item qualitative Food Frequency Questionnaire.	Snacking frequency (healthy and unhealthy); school food environment; nutritional knowledge.	Qualitative	Medium
Yip et al., 2017[130]	China	To assess the diet quality and eating behaviors of preschool children, parents’ feeding practices, and the kindergarten nutrition environment.	*n *= 302 preschool children, 30–60 months old	Physical; Sociocultural; Policy	School meals; Healthy eating learning opportunities	School environment and policy; Meal-specific; Item-specific; Individual	24-hour dietary recalls and questionnaires.	Meal types; times and locations of food consumption; cooking methods of meals consumed; acceptance of new foods or dishes; most common foods and beverages.	Quantitative	High
Yoong et al., 2015 [131]	Australia	To assess healthy food availability, pricing, promotion strategies, and variation by school characteristics in primary school canteens.	*n* = 340 school principals and *n* = 203 canteen managers at schools in New South Wales	Physical; Economic; Sociocultural; Policy	School meals; Smart snacks; Food and beverage marketing; Healthy eating learning opportunities	School environment and policy; Cafeteria environment; Meal-specific; Item-specific	Computer assisted telephone interview with canteen managers; menu audits.	Proportions of healthy/unhealthy foods; canteen operations; manager’s knowledge of healthy strategies; pricing/promotion practices.	Mixed	Low
Zive et al., 2002 [132]	USA	To describe the food environment at 24 middle schools and multiple food sources’ dietary fat contribution.	*n* = 24 middle schools	Policy	School meals	School environment and policy; Meal-specific	Bagged lunch content observation; school meal sample collection; à la carte and store data; interviews with food service staff.	Sales; nutrient analysis (i.e., grams of saturated and total fat).	Quantitative	Low

Table 2. Summary of the included studies featuring measurement tools capturing the dimensions of school food environments and the determinants of student eating behaviors. Total *n* = 101.

### Country of origin

47% (*n* = 47) of studies were conducted in the United States (Table 2). The second most studied country was Brazil (8%, *n* = 8), followed by Canada (7%, *n* = 7) and New Zealand (4%, *n* = 4). There were three or less studies from the remaining countries in Asia, Africa, South America, Europe, and Australia.

### Methods employed by measurement tools

Of the 101 studies, 60% (*n* = 61) used only quantitative methods to evaluate the school food environment. Most studies employed questionnaires and/or surveys (*n* = 65), while others incorporated menu audits (*n* = 12), dietary records (*n* = 9), sales data (*n* = 3), and/or others. Eleven studies using quantitative methods were rated as ‘high’ quality (Table 2), all of which included questionnaires and/or surveys. The most common pairing of quantitative methods were dietary records and questionnaires, and menu audits and questionnaires.

Fifteen studies (15%) used only qualitative methods to evaluate the school food environment. Interviews and focus groups were often used (*n* = 12) where students, staff, parents, and/or other stakeholders were asked about their perspectives, knowledge, and observations of the school food environment (Table 2).

There were 25 studies (25%) that took a mixed methods approach where at least one quantitative method was combined with a qualitative method to evaluate the school food environment (Table 2). Focus groups and interviews were the most commonly employed methods across mixed methods studies, and were often complemented with menu audits (Table 2). The qualitative method of research observation was also often paired with student questionnaires).

### INFORMAS dimensions of the school food environment

**Fig. 2:**
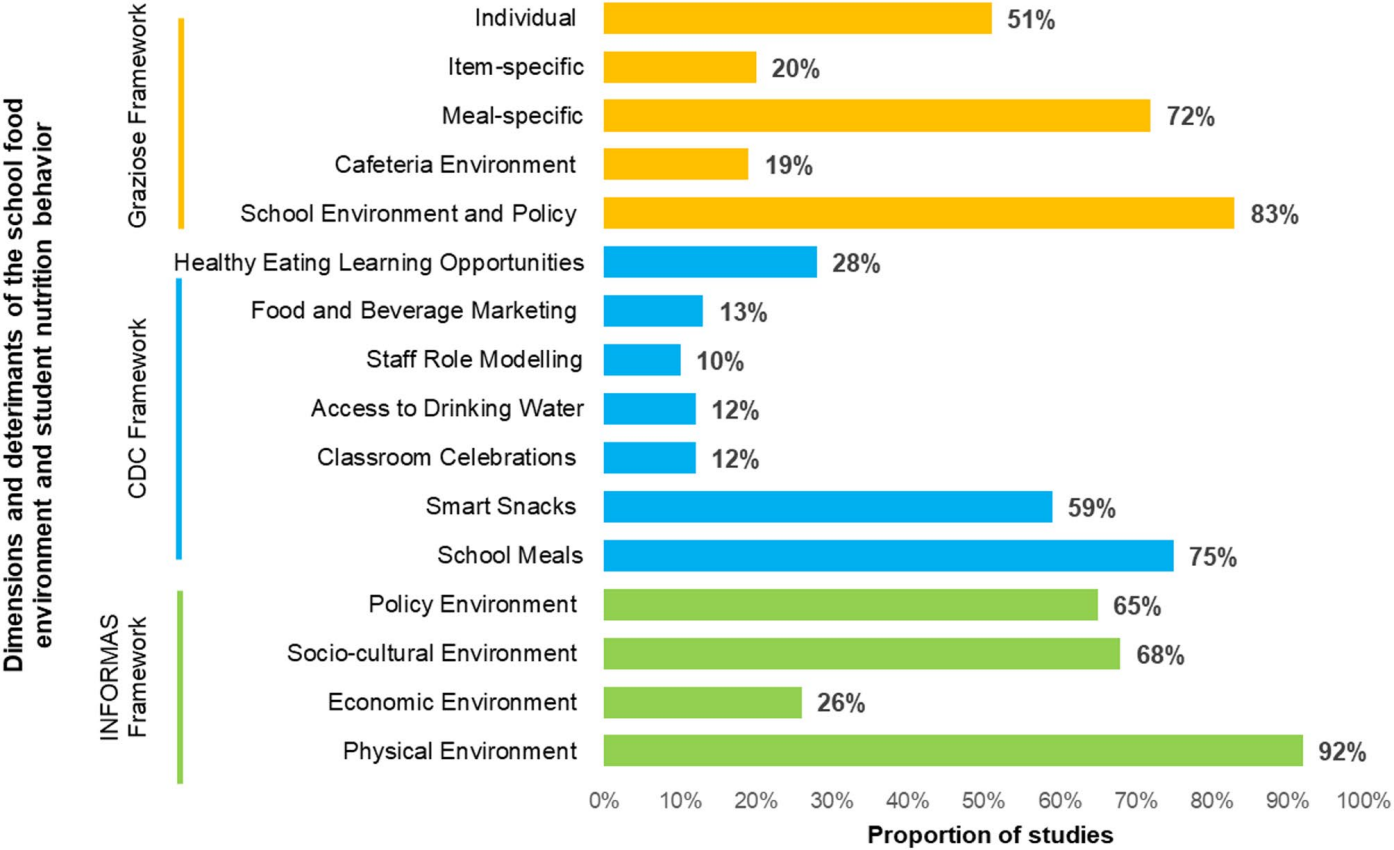
Proportion of studies that captured each dimension or determinants of school food environments and student eating behavior by framework. Total *n* = 101

Only 13% of all studies (*n* = 13) captured all four INFORMAS dimensions of the school food environment [37, 41, 42, 49, 51, 58, 71, 90, 100, 101, 116, 121, 131]. 38% (*n* = 38) measured three dimensions, 37% (*n* = 37) measured two dimensions, and 13% (*n* = 13) measured only one dimension of the school food environment. The spread of dimensions captured by measurement tools in the studies is presented in Fig. 2.

The physical dimension of the school food environment was captured by a majority (92%, *n* = 93) of the studies. 58% (*n* = 54/93) of them used quantitative methods, 16% (*n* = 15/93) used qualitative methods, and 27% (*n* = 25/93) used mixed methods. This dimension mostly captured the availability of foods and infrastructure to facilitate students’ access to healthy and safe foods at school (Table 2).

The economic dimension was the least captured, with 26% (*n* = 26) of the studies measuring financial determinants of the school food environment. Studies capturing the economic dimension evaluated and compared the prices of food items sold in school canteens or cafeterias with the prices of competitive foods (Table 2). Prices of foods available in schools were collected using questionnaires, and/or by school and food service staff surveys or interviews. Overall, studies demonstrated that the prices of unhealthy foods sold in school were lower, and thereby more affordable to the students than healthier alternatives available (Table 2).

The socio-cultural dimension of the school food environment was measured by 68% of the studies (*n* = 68). This information was widely captured using quantitative methods (54%, *n* = 37/68). The review yielded 66 studies (65%) that capture the policy dimension of the school food environment. Studies measuring the policy dimension determined the existence of policies related to school food and nutrition, as well as the adherence to and effectiveness of the policies (Table 2). Only two studies solely assessed the policy environment [124, 132].

### CDC – comprehensive framework for addressing the school nutrition environment and services

School meals were measured by 75% of the studies (*n* = 76). Smart snacks in schools, including competitive foods, were captured by 59% of the studies (*n* = 60). Classroom celebrations, events, and non-food rewards were measured by 12% of the studies (*n* = 12). Access to drinking water was also measured by 12% of the studies (*n* = 12), while staff role modelling was measured by only 10% (*n* = 10). Food and beverage marketing within schools was captured by 13% (*n* = 13) of the studies, and healthy eating learning opportunities, such as food literacy workshops and home economic classes, were measured by 28% (*n* = 28) (Table 2).

### Socioecological framework of environmental influences on elementary students’ consumption of fruits and vegetables at school lunch

Most studies investigated the school environment and policy factors (83%, *n* = 84) and meal-specific factors (72%, *n* = 73). Common meal-specific factors captured were the types of items offered within schools and the plate or serving sizes (Table 2). Cafeteria factors, such as recess times and placement, allocated time for lunch, and food delivery method (i.e., offering system vs. self-serve), were measured by only 19% of the studies (*n* = 19). Item specific factors were evaluated by 20% of the studies (*n* = 20), while individual factors, such as age, grade, gender, and overall diet quality of the study population, were captured by 51% (*n* = 52).

### Principles of Canada’s National School Food Policy 

Table 3 summarizes the principles of Canada’s National School Food Policy captured by the 7 Canadian studies included in this review. The health promoting principle was evaluated by measurement tools in all studies with a particular focus on the availability of healthy foods at schools. Whether SFPs are accessible to children and accountable to ensure they are achieving policy objectives were each evaluated by 4 out of the 7 studies. Otherwise, the remaining principles (inclusive, flexible, and sustainable) were only captured by one of 7 studies. Notably, there were no tools that evaluated all 7 principles.

**Table 3:** Principles of Canada's National School Food Policy captured by existing school food environment measurement tools in Canada

Author	Objective of Study	Principles of Canada’s National School Food Policy Captured
Black et al., 2015 [42]	To assess the integration of healthy and sustainable food initiatives in elementary and secondary schools.	Accessible, Health Promoting, Flexible, Sustainable
Callaghan et al., 2010 [48]	To evaluate the impact of a healthy vending machine pilot project.	Accessible, Health Promoting
Fitzpatrick et al., 2017 [59]	To examine the relationship between school indoor dietary environment, the surrounding school neighborhoods and adiposity in children.	Health Promoting, Accountable
Godin et al., 2019 [63]	To examine associations between Canadian adolescents’ sugar-sweetened beverage consumption and school food environment characteristics.	Accessible, Health Promoting
Masse et al., 2013 [89]	To determine whether the availability of certain foods was associated with school environment policies.	Accessible, Health Promoting, Inclusive, Accountable
McIsaac et al., 2015 [91]	To evaluate policy adherence of school lunch menus in Nova Scotia.	Health Promoting, Accountable
Stephenson et al., 2013 [110]	To evaluate of the potential impact of the Healthy Eating Guidelines Initiative on the school food environment and students’ self-reported diets.	Health Promoting, Accountable

### Quality assessment of measurement tools

Based on the quality assessment criteria, measurement tools from 17 studies (17%) were considered ‘high’ quality (Table 2). These studies were conducted in high-income countries, had large sample sizes, and most used a validated questionnaire to measure their outcomes of interest. Almost half of the tools (49%, *n* = 49) received a ‘medium’ quality rating, and a considerable number of tools (35%, *n* = 35) received a ‘low’ quality rating.

## Discussion

This systematic review provides a comprehensive evaluation of existing measurement tools used to assess school food environments and nutrition behavior at school. By analyzing 101 studies across multiple countries and methodologies, our findings highlight significant gaps in measurement coverage, methodological inconsistencies, and areas requiring further development to inform the creation of a robust school food environment audit tool for Canada’s national SFP.

Despite the growing recognition of school food environments as critical determinants of student health and nutrition behaviors [1, 133], our findings indicate substantial gaps in measurement coverage. The majority of studies focused on the physical dimension (92%), emphasizing the availability and accessibility of food within schools. This reflects ongoing research that suggest the physical environment has significant effects on one’s diet [134–136]. Labelling practices and marketing of unhealthy foods are two food policy targets endorsed by many governments to improve the physical food environment [137–139], and, in turn, the population’s diet. Similarly, implementing positive changes to the physical environment is one of the most actionable and direct strategies to improve students’ food choices and eating behavior [140, 141]. However, evidence from the recent INFORMAS Canada 2025 report highlights only 14% of schools surveyed exclusively offered healthier foods, while sugary drinks and processed foods such as cookies and pizza with processed meat were widely available [142]. Furthermore, most schools were found to be using unhealthy foods for fundraising or as rewards, and incorporating corporate-sponsored educational materials in their classes. While they can seem harmless, these indirect marketing strategies continue to shape student’s dietary preferences and behaviors, potentially undermining efforts to promote healthier eating habits in school [138]. These findings reveal critical gaps in the physical dimension of the school food environment in Canada and underscore the need for targeted design of the national SFP to promote healthier school food environments.

 Other critical dimensions, such as the economic (26%), policy (65%), and socio-cultural (68%) environments, were far less studied. This imbalance suggests that existing tools may not fully capture the structural and systemic factors influencing school food environments, including affordability, pricing strategies, and the enforcement of food policies. The economic dimension, in particular, is crucial for evaluating disparities in access to healthy food choices [143–145]. In fact, research suggests food insecurity and associated poor health outcomes are closely related to being in a low-income household [144, 145]. A study in Canada demonstrated that this is especially prominent in the school setting, where a lower socioeconomic status of children was associated with an increased risk of childhood obesity [146]. No studies in this review exclusively assessed the economic environment but rather evaluated it together with the other dimensions. This aligns with the consensus that nutrition behavior of an individual is a highly complex process involving the interplay of multiple determinants [147]. Nonetheless, pricing data captured across the studies indicate challenges in affordability for nutritionally favourable diets [34, 36, 51, 101, 105, 131], and suggest an opportunity for effective pricing strategies to make the healthier choice the easiest option for children in school. Given the importance of economic factors in determining child health and nutrition [140, 143], its consideration and representation should be prioritized in school food measurement tool development.

Overall, only 13% (*n* = 13) of the studies considered all four INFORMAS dimensions of the school food environment (Fig. 2). Evidence suggests the dimensions are interconnected and collectively exert influence on one’s nutrition behavior and nutritional status, and thus, the exclusion of any one dimension would be an incomplete evaluation [28]. For example, gathering information on what food is available through menu audits (i.e. the physical dimension), but not assessing food prices (i.e. the economic dimension), student preferences (i.e. the sociocultural dimension), and why those foods are offered in the first place (i.e. the policy dimension) limits our understanding of the school food environment and the development of an effective, tailored SFP. Therefore, school food measurement tools should capture all four dimensions.

Methodologically, most studies relied on quantitative approaches (60%), with surveys and questionnaires being the dominant tools. While these methods provide valuable insights into food availability and purchasing behaviors, they may overlook nuanced socio-cultural and policy influences. The limited use of qualitative methods (15%) and mixed-methods approaches (25%) suggests a need for more holistic evaluation strategies that incorporate stakeholder perspectives, including students, parents, and school staff, to better understand contextual barriers and facilitators to healthy eating. These findings align with the emerging recognition that research on students and stakeholders’ perceived barriers and facilitators to healthy eating is important for informing the development of effective and appropriate interventions [148–150]. Research examining school-level stakeholder perspectives provided insights into the facilitators and barriers, including lack of funding, human resources, and lack of purpose behind the programming, and implementing existing SFPs in Ontario, Canada [151, 152]. Further research on stakeholder perspectives is, therefore, needed to inform the development of an effective national SFP that aligns with the goals outlined in the Policy and stakeholder perspectives. Other strengths and weaknesses of the different measurement methods have been summarized in Supplementary Materials 4 and 5.

The purpose of applying the two additional school food environment frameworks to our review was to provide deeper insights into what information is currently being collected by existing measurement tools and to identify gaps within current research on school food environments. Collectively, we found that access to drinking water, food and beverage marketing, classroom celebrations, and item-specific factors were not measured by the majority of the studies in the review. CDC’s framework (Table 2) was an extension of CDC’s Whole School, Whole Community, Whole Child (WSCC) model designed to help shape children’s lifelong healthy eating behaviors [29]. Its primary objective was to present components of the school food environment and services to guide recommendations for school policies and practices supporting healthy diets of children. Our review suggests, however, these established components have not all been extensively evaluated in schools, and thus, focused research on school-specific food environment components is needed to inform future school food policies.

In developing their framework, Graziose demonstrated that most factors contributing to elementary students’ fruit and vegetable consumption were at the physical dimension, suggesting that the physical environment should be explored in greater depth [30]. Accordingly, the factors describing the physical environment (meal-specific factors and school environment) were the most frequently explored among studies included in our review. These findings reinforce prior research indicating aspects such as food presentation, cafeteria layout, and meal timing significantly influence students’ food choices and nutrition behavior [140, 141]. Furthermore, studies suggest that small modifications to the physical environment – such as increasing the visibility of fruits and vegetables, adjusting portion sizes, and altering the placement of healthier options – can lead to improved nutrition behaviors among students [153]. Despite the dominance of the physical environment factors in existing measurement tools, our findings also highlight the importance of individual factors such as students’ dietary preferences, eating habits, and behavioral responses to interventions. Studies capturing individual factors (51%) suggest that personal characteristics, including age, gender, and previous dietary behaviors, significantly shape food choices in school settings. Therefore, our findings support the necessity of incorporating evidence-based cafeteria infrastructure and meal service design into SFPs, while accounting for individual differences to capitalize the influence SFPs can have on children’s health.

A key objective of this review was to assess how well existing measurement tools align with the guiding principles of Canada’s National School Food Policy. Notably, the “health-promoting” characteristic was the most frequently captured, with a strong emphasis on evaluating the availability of nutritious foods in schools. However, other principles, such as inclusivity, flexibility, and sustainability, were largely overlooked in measurement efforts. The absence of tools assessing the inclusivity characteristic is particularly concerning, given the primary goal of Canada’s Policy being that all children and youth in Canada have access to nutritious at school in an inclusive and non-stigmatizing environment [10]. Furthermore, an evaluation of how culturally diverse elementary school students perceived and participated in a 2-year, universal school lunch pilot in Saskatoon, Canada cautioned against a one-size-fits-all SFP, emphasizing the need for a multi-pronged approach of health equity in designing an impactful national SFP [150]. In addition, an examination of existing provincial and territorial SFPs during the 2018/2019 school year found that accountability measures, such as transparent monitoring and evaluation systems, are critical for the success of national SFPs but remain inconsistently applied across jurisdictions [17]. In particular, previous research and national census data demonstrate that sociocultural, geographic diversity, and socioeconomic disparities remain prevalent in Canada [154, 155]. Diversity comes with many benefits; however, it is also a potential barrier to achieving equity in a national SFP. Canadian school food environments differ regionally, depending on external factors, such as the dominant demographic or culture across student populations, equity factors (i.e. socioeconomic status of neighbourhood and students), and the role of Aboriginal status as a determinant of health [11, 156]. To evaluate the Canadian school food environment and impacts of the forthcoming national SFP accurately and comprehensively, measurement tools, therefore, need to be specific to the Canadian context in recognizing the diversity of Canadian school food environments. Furthermore, in designing the national SFP, inclusivity that accounts for equity and diversity must be integrated.

A critical reflection on the three frameworks applied in this review is also warranted. The INFORMAS Framework provides a well-established, comprehensive methodology for evaluating food environments and has been validated and applied internationally [28]. However, it is not tailored to school settings, where food environments are uniquely shaped by institutional policies, curriculum, social dynamics, and the dual role of schools as both educational and food service providers [17, 25]. The CDC Frameworks and the Graziose Framework both offer more specific guidance for schools [29, 30], particularly around policy and practice, and behavioral dimensions, and provide insights into factors that influence students’ nutrition behaviors within the school context. Their limitation, however, is that both were developed for the U.S. context and may not fully reflect Canadian or other international contexts. Taken together, these three frameworks complement one another and can synergistically capture the multidimensional nature of school food environments, but each has limitations when applied in isolation.

In addition, the quality assessment of measurement tools varied considerably, with only 17% of studies employing high-quality tools that featured validated instruments, large sample sizes, and rigorous methodologies. Nearly half were rated as medium quality, while 35% were considered low quality, raising concerns about the validity of findings used to inform school food policies. While tools rated as high quality demonstrate promise, most were developed for specific contexts and would require adaptation to align with Canada’s National School Food Policy and its six guiding principles. For example, the School Food Environment Assessment Tool was developed by Black et al. to evaluate actions across six domains in Vancouver public schools, was rated as a high-quality tool in our review [42]. However, despite its methodological strengths, it is limited in its ability to capture the inclusivity dimension of SFPs. Its scoring system does not account for cultural appropriateness or responsiveness to diverse dietary needs, which are emphasized in the Policy [10]. Incorporating measures such as menu audits looking at cultural representation in food offerings, as well as interviews with students, parents, and community members to capture lived experiences, could further the tool’s potential to be leveraged in future M&E efforts and align more closely with Canada’s vision for a national SFP. Furthermore, to improve measurement reliability, future tools should prioritize validated instruments encompassing all school food environment dimensions, adopt mixed-methods approaches to integrate both quantitative and qualitative insights, and establish standardized methodologies to enhance comparability across different school settings and jurisdictions.

Our findings highlight the critical need for a robust M&E framework and subsequently, a comprehensive, tailored school food environment audit tool to support the effective implementation and evaluation of Canada’s forthcoming national SFP. This framework and tool should: (1) integrate all six guiding principles of the national policy, (2) capture multidimensional aspects of the school food environment, including the economic, socio-cultural, and policy dimensions, as well as school-specific factors, (3) be adaptable to regional and cultural variations across Canada, and (4) incorporate stakeholder perspectives to ensure inclusive and equitable program design. Such an M&E framework would provide the foundation for standardized evaluation metrics, enabling systematic data collection and assessment of key indicators such as food availability, regulatory oversight, and program accessibility. By facilitating consistent monitoring, this framework would support evidence-based decision-making and continuous policy improvement. Future research should focus on developing and validating this framework, incorporating insights from this review and expert consultation, as well as translating it into an audit tool. Additionally, longitudinal studies assessing the impact of school food policies on student nutrition behaviors, health outcomes, and academic performance will be essential in demonstrating the long-term benefits of a well-structured national SFP.

### Strengths and limitations

A major strength of this review is that a comprehensive and evidence-based search strategy of literature was used to screen, select, and evaluate studies featuring measurement tools of the school food environment. A diverse range of studies employing different methodologies (i.e. quantitative, qualitative, and mixed methods) were included in this review, and consideration of both umbrella framework and more specific, relevant frameworks were used to categorize and evaluate studies. This allowed for thorough investigation of the literature to address the research questions. However, this study has some limitations. First, our search strategy may have missed relevant studies because the search term “food environment” is coined and used primarily in North America, and other parallel terms may be used elsewhere. We limited inclusion to peer-reviewed literature and updated the search in August 2024; tools published afterward or in the grey literature were not captured. Second, although we assessed the quality of measurement tools themselves, a formal risk-of-bias or study-quality appraisal was not done for the studies. However, at least two reviewers independently screened the studies, used a predefined coding framework, and had team experts resolve any conflicts to minimize bias. Lastly, as our review aimed to gain insight to inform the development and design of a Canadian SFP M&E audit tool, discussion of other countries was included for context but not explored in-depth, as they fell outside the primary scope of this review.

## Conclusions

The results identify significant gaps and inconsistencies in current school food environment measurement tools, highlighting an urgent need for research in this field. A significant opportunity exists for policy makers in Canada to work collaboratively with researchers, school boards, growers, producers, suppliers, teachers, families, children, and communities to build a robust framework to guide the design, monitoring and evaluation of SFPs that use both federal and other funding. By addressing methodological limitations, expanding measurement coverage, and integrating best practices from international contexts, it is feasible to rapidly develop and test a standardized, comprehensive, audit tool tailored to Canada’s National School Food Policy. Such efforts will be instrumental in ensuring that Canada’s national SFP is equitable, effective, and aligned with the broader goal of promoting lifelong healthy eating habits among children and youth.

## Supplementary Information

Supplementary Material 1.

Supplementary Material 2.

Supplementary Material 3.

Supplementary Material 4.

Supplementary Material 5.

## Data Availability

All data generated or analysed during this study are included in this published article and its supplementary information files.

## Abbreviations

SFP: School food program
M&E: Monitoring and evaluation
PRISMA: Preferred Reporting Items for Systematic Reviews and Meta-Analysis
INFORMAS: International Network for Food and Obesity/non-communicable Diseases Research, Monitoring and Action Support
CDC: Centers for Disease Control and Prevention
