# Supplementary material for: Towards a comprehensive school food environment audit tool in Canada: a systematic review of school food environment measurements and nutrition determinants
Source: BMC Public Health. 2025 Oct 28;25:3636. doi: 10.1186/s12889-025-24937-w (PMC12570449; doi:10.1186/s12889-025-24937-w)
Supplement: Supplementary file 1 — Supplementary Material 1. [file 12889_2025_24937_MOESM1_ESM.docx]

**Additional Table 2.** Descriptions and definitions of INFORMAS dimensions, CDC components, and Graziose factors.

| **INFORMAS dimensions (25)** | **CDC components (29)** | **Graziose factors (30)** |
| --- | --- | --- |
| The **physical** environment (what was available?)  Availability, adequacy, and infrastructure of cafeterias, canteens, and facilities. Availability of foods and frequency of students’ purchasing periods. Menu labelling practices, if any, and presence of promotional material. Availability of vending machines and tuck shops. | **School Meals**  School meals served through the school nutrition & food program. | **School Environment and Policy Factors**  Participation in a nutrition policy or program; school-wide social marketing; nutrition education curriculum. |
| The **economic** environment (what were the financial factors?)  Type of food service employed – for profit, private, not-for-profit, etc. Price and placement of all foods available and comparison between healthy vs unhealthy foods. Student budgets and their household income. | **Smart Snacks in Schools (“Competitive Foods”)**  Foods and beverages sold during the school day outside of school meal programs; commonly referred to as "competitive foods." Includes the following subcategories: (1) in-school fundraisers; (2) à la carte foods; (3) vending machines; (4) school stores and snack bars. | **Cafeteria Environment Factors**  Food incentives (i.e. rewards); recess placement; the amount of time allocated for lunch; noise levels during lunch hours; verbal prompts (i.e. offering students healthy items in the cafeteria line); existence of a salad bar. |
| The **socio-cultural** environment (what were the attitudes and perceptions?)  Attitudes, beliefs, knowledge, and perceptions related to nutrition of students, school staff, and parents. Availability of food literacy and/or nutrition education. Prioritization of healthy food provision and students’ nutrition by school management. | **Classroom Celebrations, Events, and Non-food Rewards**  Foods and beverages given to students during classroom celebrations and other special events or as a reward for academic or behavioral achievement. | **Meal-specific Factors**  The number of items offered, portion sizes of meals and food items, the order of service, and how foods are presented to students. |
| The **policy** environment (what were the rules?)  Existence of nutrition policies and degree of adherence to policies. Elaborate regulations around food environment outlined by the employed policy. Stakeholders of existing policy and adherence monitoring efforts. Effectiveness of the existing policy. | **Access to Drinking Water**  Access to free plain drinking water for students; students must be permitted to carry reusable water bottles. | **Individual Factors**  The influence of age/grade, gender, and overall diet quality of the students. |
|  | **Staff Role Modelling**  Demonstration of healthy nutrition habits by school staff. |  |
|  | **Food and Beverage Marketing**  Marketing of food and beverage products within schools (i.e. on posters, on in-school television advertisements, placement of certain food items to make them more accessible). |  |
|  | **Healthy Eating Learning Opportunities**  Nutrition education opportunities and policies in place that support healthy learning among students; part of school curriculum. |  |
