# Supplementary material for: Towards a comprehensive school food environment audit tool in Canada: a systematic review of school food environment measurements and nutrition determinants
Source: BMC Public Health. 2025 Oct 28;25:3636. doi: 10.1186/s12889-025-24937-w (PMC12570449; doi:10.1186/s12889-025-24937-w)
Supplement: Supplementary file 2 — Supplementary Material 2. [file 12889_2025_24937_MOESM2_ESM.docx]

**Additional Table 5.** Strength(s) and limitation(s) observed across qualitative school food environment measurement tools included in the review.

| **Measurement Tool** | **n (%)** | **Strength(s)** | **Limitation(s)** |
| --- | --- | --- | --- |
| **Interviews (with students, staff, parents)** | 25 (25%) | Provides in-depth information on school food environment | Requires training, more costly. Unstructured interviews can be difficult to compare |
| **Focus Groups (with students, staff, parents)** | 6 (6%) | Provides rich insights into perceptions and experiences | Limited generalizability, potential group dynamics bias and moderator influence. Time-consuming and resource-intensive. |
| **Observation by researchers (Assessment of infrastructure, student behaviour during lunch hours, etc.)** | 12 (12%) | Provide unbiased data on school food environment | Requires training, most costly. Does not capture student perceptions (i.e. their reasons for choosing certain lunch items, why they didn't finish their food, etc.) |
| **Photovoice** | 3 (3%) | Provides visual and context-rich data on participants' experiences. | Subjectivity of responses and interpretation; costly; ethical considerations related to privacy of study participants |
