# Supplementary material for: Towards a comprehensive school food environment audit tool in Canada: a systematic review of school food environment measurements and nutrition determinants
Source: BMC Public Health. 2025 Oct 28;25:3636. doi: 10.1186/s12889-025-24937-w (PMC12570449; doi:10.1186/s12889-025-24937-w)
Supplement: Supplementary file 3 — Supplementary Material 3. [file 12889_2025_24937_MOESM3_ESM.docx]

**Additional Table 4.** Strength(s) and limitation(s) observed across quantitative school food environment measurement tools included in the review.

| **Measurement Tool** | **n (%)** | **Strength(s)** | **Limitation(s)** |
| --- | --- | --- | --- |
| **Menu audit** | 16 (16%) | Provides quantitative data on food available to students | Doesn't provide information about intake and purchasing behaviours of students |
| **Self-report dietary records completed by students (24 Hour Recall, FFQs)** | 9 (9%) | Feasible, low cost; data easily comparable | Self-assessment may be inaccurate; exacerbated by the young age of some sample groups |
| **Questionnaires and surveys (completed by school staff and/or parents)** | 65 (64%) | Feasible, low cost; data easily comparable | Self-assessment may be inaccurate |
| **Sales data collection** | 3 (3%) | Provides quantitative data on foods purchased by students | Does not provide information on how much of the food was eaten and why certain foods were chosen over others |
