# Supplementary material for: Towards a comprehensive school food environment audit tool in Canada: a systematic review of school food environment measurements and nutrition determinants
Source: BMC Public Health. 2025 Oct 28;25:3636. doi: 10.1186/s12889-025-24937-w (PMC12570449; doi:10.1186/s12889-025-24937-w)
Supplement: Supplementary file 4 — Supplementary Material 4. [file 12889_2025_24937_MOESM4_ESM.docx]

**Additional Table 1.** Search concepts and terms used to identify school food measurement tools used in peer-reviewed literature.

| **Research Concept** | **Search Terms** | **Search Strategy** |
| --- | --- | --- |
| Food environments | food | (food OR “school food environment” OR “school nutrition environment” OR nutrition) |
|  | school food environment |  |
|  | school nutrition environment |  |
|  | nutrition |  |
| Schools | school* | (school* OR student* OR lunch* OR cafeteria* OR “school food environment” OR “school nutrition environment") |
|  | student* |  |
|  | lunch* |  |
|  | cafeteria* |  |
|  | school food environment |  |
|  | school nutrition environment |  |
| Measurement tools | assess* | (assess* OR measur* OR “measurement tool*” OR “assessment tool*” OR evaluat*) |
|  | measur* |  |
|  | measurement tool* |  |
|  | assessment tool* |  |
|  | evaluat* |  |
| **Combined search:**  (food OR “school food environment” OR “school nutrition environment” OR nutrition) AND (school* OR student* OR lunch* OR cafeteria* OR “school food environment” OR “school nutrition environment") AND (assess* OR measur* OR “measurement tool*” OR “assessment tool*” OR evaluat*) | | |
