# Supplementary material for: Towards a comprehensive school food environment audit tool in Canada: a systematic review of school food environment measurements and nutrition determinants
Source: BMC Public Health. 2025 Oct 28;25:3636. doi: 10.1186/s12889-025-24937-w (PMC12570449; doi:10.1186/s12889-025-24937-w)
Supplement: Supplementary file 5 — Supplementary Material 5. [file 12889_2025_24937_MOESM5_ESM.docx]

**Additional Table 3.** Criteria and ratings for quality assessment of the school food environment measurement tools^1^.

| **Criteria for Assessment** | **Description** | **Rating** |
| --- | --- | --- |
| **Comprehensiveness** | The ability of the methods and tools to adequately capture key aspects of food provision. The proportion of the four INFORMAS dimensions to categorize the methods used to measure the school food environment. | **Low:** only 1 dimension was included **Medium:** 2-3 dimensions were included **High:** all 4 dimensions were included |
| **Generalizability** | The degree to which the results of the study are generalizable to other groups of people or contexts. | **Low:** methods and tools are mostly country-specific **Medium:** applicable in other countries/contexts  **High:** applicable globally |
| **Relevance** | The degree to which the methods of the study are relevant and accurate to the people and situations assessed by the studies. The criterion takes into account the following aspects: the food environment is assessed by one or more than one measurement method, measurement tools are pre-tested; the sample is representative. | **Low:** meets 1 of 3 sub-criteria or does not meet any sub-criteria  **Medium:** meets 2 of 3 sub-criteria **High:** meets 3 of 3 sub-criteria |
| **Feasibility** | The ease and practicality of applying the methods and tools. The following aspects should be taken into account for the assessment: easy to administer, and interpretable. | *****assessed based on authors' judgement by comparing across studies **Low:** low feasibility relative to other studies  **Medium:** medium feasibility relative to other studies  **High:** high feasibility relative to other studies |
| **Overall Assessment** | The overall quality of the methods and tools. | **Low:** two or more 'Low' ratings for any of the assessment criteria **Medium:** three 'High' + one 'Low' rating OR four 'Medium' OR two 'High' + one 'Medium' + one 'Low' OR one 'High' + three 'Medium' OR three 'Medium' + one 'Low' OR one 'High' + two 'Medium' + one 'Low'  **High:** four 'High' OR three 'High' + one 'Medium' OR two 'High' + two 'Medium' |

^1^O’Halloran S, Eksteen G, Gebremariam M, Alston L. Measurement methods used to assess the school food environment: A systematic review. International Journal of Environmental Research and Public Health. 2020;17(5):1623. doi:10.3390/ijerph1705162
